# Beneficial Effects of Dietary Probiotics Mixture on Hemato-Immunology and Cell Apoptosis of *Labeo rohita* Fingerlings Reared at Higher Water Temperatures

**DOI:** 10.1371/journal.pone.0100929

**Published:** 2014-06-30

**Authors:** Sipra Mohapatra, Tapas Chakraborty, Ashisa K. Prusty, Kurchetti PaniPrasad, Kedar N. Mohanta

**Affiliations:** 1 Laboratory of Bioresource, NIBB, Okazaki, Japan; 2 Division of Molecular Environmental Endocrinology, NIBB, Okazaki, Japan; 3 South Ehime Fisheries Research Center, Ehime University, Ainan, Japan; 4 Project Directorate for Farming System Research, Meerut, India; 5 Central Institute of Fisheries Education, Mumbai, India; 6 Central Institute of Freshwater Aquaculture, Bhubaneswar, India; Louisiana State University School of Veterinary, United States of America

## Abstract

Probiotics play an important role in growth increment, immune enhancement and stress mitigation in fish. Increasing temperature is a major concern in present aquaculture practices as it markedly deteriorates the health condition and reduces the growth in fish. In order to explore the possibilities of using probiotics as a counter measure for temperature associated problems, a 30 days feeding trial was conducted to study the hemato-immunological and apoptosis response of *Labeo rohita* (8.3±0.4 g) reared at different water temperatures, fed with or without dietary supplementation of a probiotic mixture (PM) consisting of *Bacillus subtilis*, *Lactococcus lactis* and *Saccharomyces cerevisiae*) (10^11^ cfu kg^−1^). Three hundred and sixty fish were randomly distributed into eight treatment groups in triplicates, namely, T1(28°C+BF(Basal feed)+PM), T2(31°C+BF+PM), T3(34°C+BF+PM), T4(37°C+BF+PM), T5(28°C+BF), T6(31°C+BF), T7(34°C+BF) and T8(37°C+BF). A significant increase (P<0.01) in weight gain percentage was observed in the probiotic fed fish even when reared at higher water temperature (34–37°C). Respiratory burst assay, blood glucose, erythrocyte count, total serum protein, albumin, alkaline phosphatase and acid phosphatase were significantly higher (P<0.01) in the probiotic fed groups compared to the non-probiotic fed groups. A significant (P<0.01) effect of rearing temperature and dietary probiotic mixture on serum myeloperoxidase activity, HSP70 level and immunoglobulin production was observed. Degree of apoptosis in different tissues was also significantly reduced in probiotic-supplemented groups. Hence, the present results show that a dietary PM could be beneficial in enhancing the immune status of the fish and also help in combating the stress caused to the organism by higher rearing water temperature.

## Introduction

Alarming rise in sea temperature caused by global warming is thought to negatively affect the wild capture fisheries as well as the world aquaculture production [1]. Temperature is known to be the most critical factor in the development, growth, nutrient utilization, metabolism and immunity of many organisms including fish [2]. Although reported to survive between 18.3 and 37.8°C, without any mortality [3], carps, including *Labeo rohita*, show better growth and good metabolic functions at an ideal water temperature range of 24–28°C [4], [5]. Recently, much emphasis is laid on the study of the effects of rising temperature on the immune status of aquatic animals, and the possible ways of ameliorating the health of the fish under such environmental conditions [6].

Several studies have reported that immunostimulants and other biological factors such as probiotics, can trigger the defense system, even under stressful conditions, thereby reducing the deleterious effects caused by various biological, chemical and physiological stress [6], [7], [8], [9]. Probiotics, the beneficial microbial population, are considered to enhance the immunity of fish under stressful environmental conditions, by modulating the gut colonization of the probiotic bacterial strains, and production of antibodies, acid phosphatase, lysozyme and anti-microbial peptides [10], [11.12], [13], [14], [15].

Apoptosis, a molecularly regulated process of cell death, is known to be induced by a range of environmental, physical or chemical stresses, and is characterized by a sequence of precisely regulated events that culminate in the self-destruction of a cell. Induction of the stress responsive heat shock protein (HSP) 70, may also accelerate apoptosis in various fish tissues [16], [17]. HSPs are also considered to be the key markers for fluctuating temperature [18] and it has been shown that the protective mechanism of an animal, influenced by normal intestinal micro-flora commonly taken as probiotics, is due to an increase in the levels of the putative protective HSP under stress conditions [19]. However, till date, the beneficial effects of probiotics on higher temperature and associated anomalies in fish have not been studied. Therefore, the present work was designed to investigate the effects of a probiotic-enriched diet in enhancing the immune status of the fish by analyzing their growth, hemato-immunological parameters and progression of cell death at different rearing water temperatures.

## Materials and Methods

All experiments were conducted in accordance with the legal requirements of India and Japan. The Institutional Animal Care and Use Committee at the Indian Council of Agricultural Research (ICAR), Ministry of Agriculture, Government of India, New Delhi, approved all procedures and protocols related to treatment and maintenance of the animals.

### Experimental Fish

*L. rohita* fingerlings were procured from Palghar fish farm, Maharashtra, India, and transported to the wet laboratory of Central Institute of Fisheries Education (CIFE), Mumbai, India, with the provision of continuous aeration. The fish were subjected to salt treatment (1%) for 5 min to ameliorate the handling stress and then acclimatized to the laboratory conditions for 15 days. During the acclimatization period, the fish were fed twice a day with a basal formulated diet (350 g protein kg^−1^ diet and 17.2 MJ kg^−1^ dietary gross energy) [20].

### Water Quality Parameters

Water quality parameters were in the range of pH 7.4–7.6, dissolved oxygen 5.8–6.9 mg L^−1^, free carbon dioxide 1.9–2.7 mg L^−1^, total hardness 156–185 mg L^−1^, ammonia-N 0.14−0.37 mg L^−1^, nitrite-N 0.06±0.13 mg L^−1^ and nitrate-N 0.03±0.14 mg L^−1^ throughout the experimental period [21]. While the water temperature was recorded twice daily at 06:00 and 14:30 h, the other parameters were measured every 15 day intervals. All the above mentioned water quality parameters (except water temperature) during the entire experiment period were found to be in the optimum range for fish rearing [22].

### Experimental Design

Three hundred and sixty uniformly sized fingerlings (average weight 8.3±0.4 g) were randomly distributed in eight treatment groups (T1–T8) with three replicates (stocking density was maintained at 15 fish/300 L of rearing water) following a completely randomized design. Using a digital thermostat, the temperatures were gradually increased by 1°C per day from the initial water temperature (28°C) to the target temperatures (31, 34 and 37°C) [23]. The temperature acclimatization was initiated in such a way that all the treatments reached the target temperature on the same day. After reaching the desired temperatures, fish were fed with the experimental diets for the next 30 days. The different experimental temperatures the fish were kept at (including the ambient temperature 28°C for *L. rohita*, which was used as control group) were T1 & T5 (28°C), T2 & T6 (31°C), T3 & T7 (34°C) and T4 & T8 (37°C). After the acclimatization period, treatments T1, T2, T3 and T4 were fed with the basal diet supplemented with a combination of three probiotics (*B. subtilis, L. lactis* and *S. cerevisiae*) (1∶1∶1) (10^11^ cfu kg^−1^) whereas the other groups (T5, T6, T7 and T8) were fed with basal diet (without probiotics) for an experimental period of 30 days. Feed was given at 2.5% of body weight, twice a day at 8:00 and 18:00 h under normal light regime (light/dark: 12/12 h). Unconsumed feed and faecal matters were siphoned out daily and nearly 50% water was exchanged daily throughout the experiment period.

### Preparation of Probiotic Microorganisms

Pure strains of *B. subtilis, L. lactis* and *S. cerevisiae* were purchased from the Microbial Type Culture Collection and Gene Bank (MTCC), Chandigarh, India and maintained at 4°C in the laboratory. Subsequently, *B. subtilis*, *L. lactis* and *S. cerevisiae* were grown in Brain Heart Infusion (BHI), de Man Rogosa and Sharpe (MRS) and Yeast Extract Peptone Dextrose (YEPD) agar medium (Himedia), respectively. These freshly grown pure inoculums of probiotic strains were inoculated into a conical flask containing the respective growth medium and kept at 30°C in a shaking incubator for 24 h. The cultures were centrifuged at 10,000 g for 15 min at 4°C. The supernatant was discarded and the pellets were washed and re-centrifuged four times in phosphate buffer saline (PBS; pH 7.2), and subsequently quantified by spread plate method in order to determine the number of colony forming units (cfu). A growth curve was established for each probiotic by OD_600_ measurements of the broth cultures, in order to determine the concentration of probiotic to be added to the feed. The three probiotics were added together in equal proportions (1∶1∶1) to make a final concentration of 10^11^ cfu kg^−1^ feed [9], [15]. All feed ingredients were mixed properly, steamed for 20 min and cooled. Afterwards, the required mixture of probiotic culture (re-suspended in PBS) was mixed into the basal feed and made into pellets.

### Growth Indices

The growth of *L. rohita* fingerlings were assessed in terms of % weight gain, at the end of the experiment. The experimental fish from each tank were weighed collectively at 7 day intervals to monitor the growth of the fish. The % weight gain was calculated based on standard formula:

% Weight gain  =  [(final weight – initial weight)/(initial weight)] ×100

### Hematological Parameters

At the end of the feeding trial, four fish from each replicate tank of the different experimental groups were anaesthetized using clove oil (50 µl L^−1^) and blood was drawn from the caudal vein using a 24 gauge micro syringe coated with 2.7% EDTA solution. The blood collected from these four fishes was pooled together, and used as one biological replicate. Three biological replicates were prepared, one from each treatment replicate. The blood was transferred to a test tube coated with EDTA, and stored at −30°C until use.

The Nitroblue Tetrazolium Assay (NBT) was performed following a modified standard protocol [24] to measure the superoxide ion production. Briefly, blood (50 µL) was added to the wells of “U” bottom microtitre plates and incubated at 37°C for 1 h to facilitate the adhesion of the cells. Then the supernatant was removed and the loaded wells were rinsed three times with PBS. After rinsing, 50 µL of 0.2% NBT was added to each well before incubating the plates for 1 h. The cells were then fixed with 100% methanol for 3 min and again washed three times with 30% methanol before air-drying the plates. 60 µL 2N potassium hydroxide and 70 µL dimethyl sulfoxide were added to each well to dissolve the formazon blue precipitate formed. The O.D. of the turquoise blue colored solution was then read using an ELISA reader at a wavelength of 540 nm.

Blood glucose was estimated by the method of Nelson and Somogyi [25] and as modified by Oser [26]. Blood was deproteinized with zinc sulfate and barium hydroxide, filtered and the filtrate was taken for glucose estimation. The supernatant was collected in a test tube and alkaline copper sulfate was added. The test tubes were placed in a boiling water bath for 20 min. Arsenomolybdate reagent was added to the test tubes after cooling and the absorbance was recorded at 540 nm.

The Total Erythrocyte Count (RBC) and the Total Leucocyte Count (WBC) was determined using a Neubauer-type hemacytometer. Toission's solution and Turk's solution were used to dilute blood for RBC and WBC, respectively. Blood (20 µL) was added to glass vials containing 3980 µL of corresponding diluting solution and the mixture was shaken well to suspend the cells uniformly in the solution. The number of cells was counted using a haemocytometer.

Number of cells ml^−1^ =  (Number of cells counted in haemocytometer X dilution)/(area counted X depth of solution).

### Immunological Parameters

For serum collection, another four fish were sampled from each replicate tank of the different experimental groups. Blood was drawn without using any anticoagulant and collected in an eppendorf tube. The tubes were kept in an inclined position for 2 h at room temperature, allowing the blood to clot. The samples were then centrifuged in a cooling centrifuge at 3000 g for 5 min. The serum from all four fish of one treatment replicate were pooled together in a dry eppendorf, and used as one biological replicate. Three biological replicates were prepared, one from each treatment replicate. The serum was stored at −20 °C until use.

Qualigens kits (Qualigens Fine Chemicals, Mumbai, India) were used to analyze the different serum parameters. Serum globulin was calculated by deducting the albumin value from the total protein value. Alkaline phosphatase (ALP) and Acid phosphatase (ACP) contents were calculated using the Qualigens kit (Qualigens Diagnostics, Mumbai, India). Serum HSP was analyzed using the Anti-Human HSP 70 (Total) ELISA kit (Stressgen, USA) [27].

The serum myeloperoxidase (MPO) activity was measured according to Quade and Roth [28] with minor modification. About 10 µL of serum were diluted with 90 µL of Hank's balanced salt solution (HBSS) without Ca^2+^ or Mg^2+^ in 96-well plates. Then 35 µL of 20 mM 3, 3′, 5, 5′- tetramethylbenzidine hydrochloride (TMB) (Himedia, India) and 5 mM H_2_0_2_ (Qualigens) (both substrates of MPO, freshly prepared on the same day) were added. The color change reaction was stopped after 2 min by adding 35 µL of 4 M sulfuric acid (H_2_S0_4_). The O.D. was read at 450 nm using a microplate reader (µQuant, Universal Microplate Spectrophotometer, Northstar Scientific, UK).

The immunoglobulin level in the serum was determined by the ELISA assay [29] with slight modification. Briefly, serum samples were serially diluted in 1X Tris Buffered Saline (TBS) pH 7.4 (50 µL/well) and used to coat the 96 well flat bottomed ELISA plates (Tarsons, India). After 3 h of incubation at room temperature (25±1°C), the plates were washed three times with TBST (TBS containing 0.05% Tween 20) at 5 min intervals. Blocking reagent (5% skimmed milk powder in TBS) was added (100 µl per well) and incubated at 4°C over night. The plates were washed again with TBST for 3 times at 5 min intervals. Subsequently, 50 µl anti-rohu Ig rabbit serum (at a dilution of 1∶5000 as earlier determined by checkerboard titration method) were added to all the wells and the plates incubated for 2 h at room temperature. The plates were washed again with TBST as described above. Subsequently, goat anti-rabbit IgG HRPO (horseradish peroxidase) conjugate (Genei, Bangalore, India) was added to each well at a 1∶2000 dilution as specified by the manufacturer. After 2 h of incubation at room temperature, the plates were washed with TBST, prior to adding 100 µL of substrate, TMB/H_2_O_2_ (Genei, Bangalore, India) all each well. The color reaction was stopped by adding 1N H_2_SO_4_ (50 µL/well) immediately to all wells. The absorbance was read at 450 nm using an ELISA reader.

### Cell Death Analysis

Four anesthetized fish from each replicate tank of the different experimental groups were sacrificed (in accordance with the Animal Ethics Committee, ICAR, New Delhi, India) and fresh samples of liver, gill and kidney were immediately fixed in 10% neutral buffer formalin (NBF). The tissue samples were embedded in paraffin wax and cut into serial 5 µm sections. Cell death analysis was carried out using the *in situ* cell death kit (Roche, USA) following manufacturer's protocol. Additionally, the slides were counter-stained with Hoechst dye to visualize the nucleus. The stained slides were examined and photographed under a Nikon (A1R-si) confocal microscope. The total numbers of fluorescence positive apoptotic cells in a group of 1000 Hoechst positive cells were counted in each of 10 randomly selected areas (fixed) per section. The mean data from 10 continuous sections of 4 fish (from the same treatment replicate) were used for calculating the biological average apoptotic cell count. The mean data generated from three such biological averages were graphically presented as relative apoptotic cell population in different tissues.

### Statistical Analysis

Data were analyzed by two-way analysis of variance (ANOVA) and the significant difference between the treatment means was determined by Duncan's Multiple Range Test (DMRT) using SPSS (Statistical Package for Social Sciences, Version 16.0). A significance level of P<0.01 was used.

## Results

### Growth

**Figure 1:**
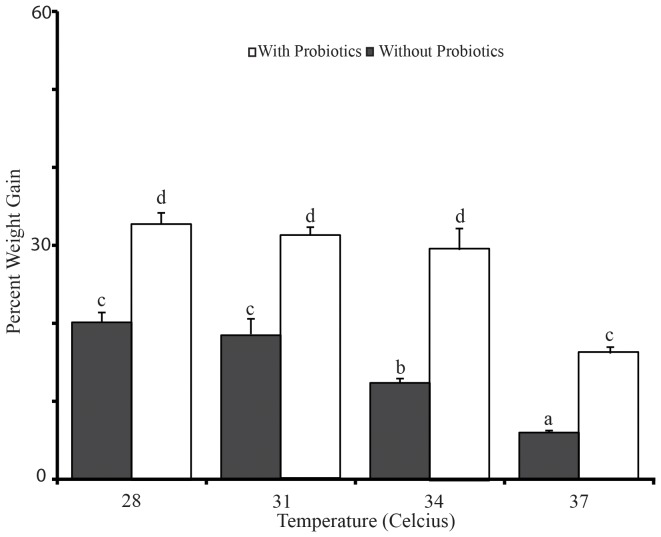
Effect of temperature and probiotic supplementation on percentage body weight gain of *Labeo rohita* fingerlings at the completion of the experiment. Significance between the treatments was determined by Duncan's Multiple Range Test (DMRT) using SPSS (Version 16.0). Similar alphabets (a,b,c,d) denotes no significance at P<0.01. Each value represents Mean ±SE of three biological replicates (n = 3), each replicate prepared from four fish. Briefly, data from four fish of one treatment replicate were averaged and used as one biological replicate. Similarly, three biological replicates (n = 3) were prepared, each from one treatment replicate.

The different levels of temperature showed a significant difference (P<0.01) on the % body weight gain of *Labeo rohita* fingerlings (Fig. 1). Significant increase (P<0.01) in % weight gain was noticed in the group maintained at 28 and 31°C, while significant reduction (P<0.01) was observed in fish maintained at 34 and 37°C, respectively. Interestingly, the probiotic fed fish showed better growth than the non-probiotic fed fish. Significant (P<0.01) interaction effect of probiotic and temperature level was also observed on the % weight gain of the fingerlings. No mortality was observed in any of the tanks at any point of the experimental period.

### Hematological Studies

The respiratory burst activity (NBT reduction) of neutrophils of *L. rohita* juveniles of different experimental groups is presented in Table 1. There was a significant effect (P<0.01) of different temperature on respiratory burst activity. NBT activity exhibited a decreasing trend with an increase in rearing temperature. However, there was no significant (P>0.01) effect of the interaction of probiotics and temperature on the respiratory burst activity. On the other hand, blood glucose levels showed a completely opposite pattern (Table 1). Fish fed with probiotic supplemented diet had lower blood glucose levels in comparison to the non-supplemented groups. Blood glucose levels were also significantly (P<0.01) affected by probiotics, temperature and their interactions. Dietary probiotics significantly (P<0.01) affected the total erythrocyte count with the highest count recorded in T1 group (2.01±0.27) (Table 1). The total erythrocyte count decreased significantly (P<0.01) with increasing temperatures. Significant (P<0.01) interactive effect of probiotics and temperature was noticed for the total erythrocyte count. The total leucocyte count was significantly (P<0.01) reduced in the probiotic fed fish compared to non-probiotic fed fish. WBC count was also significantly (P<0.01) affected by the interaction of probiotics and temperature.

**Table 1:** Effect of temperature, multispecies probiotics mixture (*B. subtilis, L. lactis* and *S. cerevisiae*) and their interaction on the blood parameters of *L. rohita* fingerlings.

TREATMENT	NBT (A_540_)	Blood glucose (mg 100 ml^−1^)	RBC (X 10^6^ cells µl^−1^)	WBC (X 10^3^ cells µl^−1^)
**PROBIOTICS LEVEL**				
** With**	0.34±0.02^a^	85.26±0.47^b^	1.81±0.18^a^	2.49±0.21^b^
** Without**	0.19±0.02^b^	97.16±2.48^a^	0.71±0.20^b^	3.03±0.12^a^
** P-value**	0.000	0.000	0.000	0.000
**TEMPERATURE LEVEL**				
** 28°C**	0.32±0.02^a^	81.49±0.23^d^	1.54±0.16^a^	2.46±0.08^b^
** 31°C**	0.31±0.02^a^	86.46±0.66^c^	1.36±0.14^ab^	2.77±0.15^ab^
** 34°C**	0.26±0.01^b^	92.87±1.89^b^	1.20±0.10^bc^	2.75±0.04^ab^
** 37°C**	0.19±0.01^c^	104.03±3.8^a^	0.96±0.06^c^	3.06±0.13^a^
** P-value**	0.000	0.000	0.003	0.010
**TEMPERATURE X PROBIOTICS**				
** T1**	0.38±0.01	82.22±0.23^d^	2.01±0.27^a^	2.52±0.09^c^
** T2**	0.38±0.02	83.93±0.11^d^	1.95±0.22^a^	2.53±0.14^c^
** T3**	0.34±0.01	85.58±0.23^d^	1.81±0.13^a^	2.43±0.07^c^
** T4**	0.26±0.01	89.33±0.18^c^	1.48±0.11^b^	2.46±0.20^c^
** T5**	0.25±0.01	80.75±0.16^e^	1.06±0.03^bc^	2.39±0.33^b^
** T6**	0.23±0.01	88.99±0.12^c^	0.77±0.20^cd^	3.00±0.16^b^
** T7**	0.17±0.02	100.17±0.18^b^	0.59±0.06^d^	3.07±0.02^b^
** T8**	0.12±0.02	118.73±0.25^a^	0.43±0.03^d^	3.66±0.02^a^
** P-value**	0.046	0.000	0.000	0.000

Data expressed as Mean ± SE (n = 3). Different superscripts in each column under each sub headings vary significantly (P<0.01).

T1 (28°C+BF (Basal feed) +PM (Probiotic mixture)), T2 (31°C+BF+PM), T3 (34°C+BF+PM), T4 (37°C+BF+PM), T5 (28°C+BF), T6 (31°C+BF), T7 (34°C+BF) and T8 (37°C+BF).

### Immunological Observations

Serum characteristics of fish from the different treatment groups were analyzed and presented in Table 2. Serum total protein and globulin contents were significantly (P<0.01) reduced with increasing rearing temperatures. Significant increased values (P<0.01) of total protein and globulin were observed in the treatments fed with probiotic incorporated diets. Serum albumin to globulin ratio (A/G ratio) was significantly (P<0.01) reduced in fish fed with the probiotic incorporated diet and increased with elevated water temperature. Increasing temperature level had a significant (P<0.01) effect on the serum ALP and ACP content. However, reduced ALP and ACP contents were recorded in fish fed with probiotic supplemented diet. A significant (P<0.01) increase in the serum MPO activity was noticed in the probiotic fed fish. The serum MPO activity showed a significant difference (P<0.01) in the interaction of temperature and probiotics, with the highest value being recorded in T1 (4.08±0.26).

**Table 2:** Effect of temperature, multispecies probiotics mixture (*B. subtilis, L. lactis* and *S. cerevisiae*) and their interaction on the serum parameters of *L. rohita* fingerlings.

TREATMENT	Total protein (g dl^−1^)	Albumin (g dl^−1^)	Globulin (g dl^−1^)	A:G ratio	ALP (IU l^−1^)	ACP (IU l^−1^)	HSP (nano g ml^−1^)	MPO (A_450_)	IgM (OD)
**PROBIOTICS LEVEL**									
** With**	2.49±0.03^a^	0.79±0.01	1.70±0.04^a^	0.47±0.05^b^	133.24±1.58^b^	22.25±1.07^b^	5.86±0.06^b^	3.02±0.39^a^	0.22±0.01^a^
** Without**	2.06±0.09^b^	0.77±0.02	1.28±0.08^b^	0.64±0.04^a^	155.90±2.11^a^	33.94±0.96^a^	14.70±0.41^a^	0.78±0.28^b^	0.10±0.02^b^
** P-value**	0.000	0.100	0.001	0.000	0.000	0.000	0.000	0.000	0.000
**TEMPERATURE LEVEL**									
** 28°C**	2.47±0.05^a^	0.76±0.02	1.71±0.06^a^	0.44±0.02^b^	130.75±3.77^c^	23.56±1.55^c^	5.49±1.48^d^	2.54±0.27^a^	0.19±0.01^a^
** 31°C**	2.39±0.07^ab^	0.78±0.01	1.61±0.07^ab^	0.49±0.02^ab^	131.16±4.29^c^	24.75±3.05^c^	7.46±2.44^c^	1.90±0.20^b^	0.20±0.01^a^
** 34°C**	2.22±0.12^ab^	0.78±0.02	1.41±0.12^ab^	0.59±0.06^ab^	150.97±1.91^b^	29.20±0.43^b^	12.04±0.03^b^	1.81±0.15^b^	0.14±0.01^b^
** 37°C**	2.00±0.15^b^	0.81±0.02	1.23±0.14^b^	0.67±0.07^a^	165.40±3.27^a^	34.88±1.53^a^	16.13±1.37^a^	1.37±0.02^c^	0.12±0.00^c^
** P-value**	0.000	0.011	0.000	0.003	0.000	0.000	0.000	0.000	0.000
**TEMPERATURE X PROBIOTICS**									
** T1**	2.58±0.03^a^	0.74±0.01	1.83±0.03^a^	0.41±0.01^b^	125.12±0.99^e^	21.01±1.26^d^	5.67±0.03^f^	4.08±0.26^a^	0.25±0.00^b^
** T2**	2.54±0.03^a^	0.75±0.01	1.77±0.03^ab^	0.44±0.01^b^	123.14±0.94^e^	21.22±0.62^d^	5.78±0.02^de^	3.01±0.17^b^	0.27±0.00^a^
** T3**	2.47±0.02^a^	.077±0.01	1.67±0.02^bc^	0.48±0.01^b^	136.12±1.83^d^	22.91±0.30^d^	5.93±0.02^d^	2.90±0.09^b^	0.20±0.01^c^
** T4**	2.34±0.02^bc^	0.78±0.02	1.54±0.04^de^	0.53±0.03^b^	148.57±2.97^c^	23.85±0.60^d^	6.07±0.14^de^	2.10±0.20^c^	0.17±0.01^d^
** T5**	2.36±0.02^b^	0.79±0.04	1.60±0.05^cd^	0.48±0.04^b^	136.37±1.02^d^	26.10±1.00^c^	5.31±0.07^f^	1.00±0.01^d^	0.13±0.00^e^
** T6**	2.24±0.01^c^	0.81±0.02	1.45±0.01^e^	0.54±0.01^b^	139.18±1.37^d^	28.28±0.64^c^	9.14±0.11^c^	0.79±0.03^d^	0.13±0.00^e^
** T7**	1.96±0.03^d^	0.81±0.05	1.15±0.02^f^	0.71±0.06^a^	165.81±1.42^b^	35.49±0.53^b^	18.15±0.05^b^	0.71±0.02^d^	0.09±0.00^f^
** T8**	1.66±0.03^e^	0.81±0.03	0.92±0.01^g^	0.82±0.04^a^	182.23±0.27^a^	45.90±2.96^a^	26.18±0.03^a^	0.63±0.02^d^	0.07±0.00^g^
** P-value**	0.000	0.056	0.000	0.000	0.000	0.000	0.000	0.000	0.000

Data expressed as Mean ±SE (n = 3). Different superscripts in each column under each sub headings vary significantly (P<0.01).

T1 (28°C+BF (Basal feed) +PM (Probiotic mixture)), T2 (31°C+BF+PM), T3 (34°C+BF+PM), T4 (37°C+BF+PM), T5 (28°C+BF), T6 (31°C+BF), T7 (34°C+BF) and T8 (37°C+BF).

The probiotic fed groups showed nearly two times lower HSP70 activity than non-probiotic fed group (Table 2). It was observed that the HSP70 production in the body increased significantly (P<0.01) with the rise in water temperature. The antibody production, in terms of IgM production, was also significantly (P<0.01) increased in the probiotic fed fish. However, antibody production decreased significantly (P<0.01) with the increase in temperature. Significant interaction (P<0.01) effect of probiotics and temperature on IgM production in the fish was also observed.

### Cell Death Analysis

**Figure 2:**
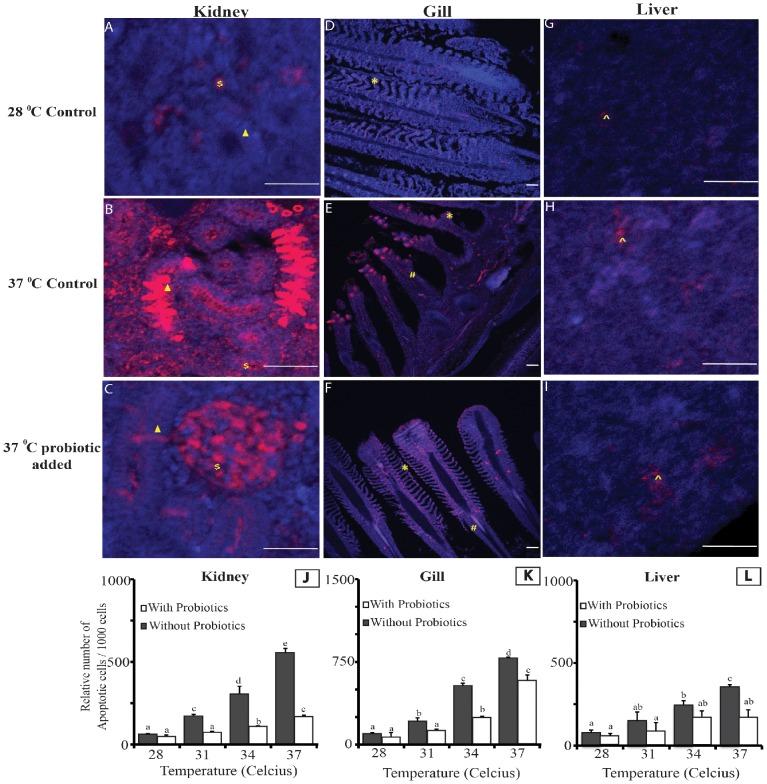
Analysis of the effects of temperature and probiotic supplementation on apoptosis in Kidney (A–C), Gill (D–F) and Liver (G–I). The apoptotic cells are represented by red color while blue color represents the juxtaposed cell nucleus. Note: Different cell types i.e. nephrons, interstitial cells of kidney, primary gill lamellae, secondary gill lamellae and apoptotic hepatic cells are respectively marked with ▴, $, #, * and ∧. Bar length- 50 micron. J–L shows the relative apoptotic cell density in the kidney, gill and liver at different temperature. Each value represents Mean ±SE of three biological replicates (n = 3), each replicate prepared from four fish. Briefly, the mean data from 4 fish (from same treatment replicate) were used for calculating the biological average apoptotic cell count. The average data generated from three such biological averages (n = 3) were used for statistical analysis. Significance between the treatments was determined by Duncan's Multiple Range Test (DMRT) using SPSS (Version 16.0).

The concentration of apoptotic cells in different organs with respect to change in water temperature in different treatment groups are presented in Figs 2A-L. Similar to other parameters tested in this study, the highest temperature group (T8) depicted the highest density of apoptotic cell in gills, kidney and liver sections (Figs. 2A–K). Amongst all tissues that were tested, gill sections showed the highest density of apoptotic cells per 1000 cells counted (Figs. 2J–L). Probiotic supplementation helped to significantly reduce (p<0.01) the rate of apoptosis in kidney and liver at higher temperature. However, no distinct differences (P>0.01) were observed between the gills of fish from probiotic and non-probiotic fed groups (Fig. 2K).

## Discussion

Fish are poikilothermic vertebrates that inhabit aquatic ecosystem and are most susceptible to seasonal and diurnal variations in water temperature. Temperature is known to regulate the growth and other physiological and biochemical functions of fish [23]; [30]; [31]. The present study clearly shows that elevated water temperature alters several hemato-immunological parameters, enhances the synthesis of stress related proteins production and induces apoptosis, ultimately resulting in an overall reduction of fish growth. A three species combination of probiotics, namely *B. subtilis*, *L. lactis* and *S. cerevisiae*, was used to overcome the temperature-associated stress, which also substantially improved the fish growth.

Any kind of stress causes intensive gluconeogeneis and thus increases blood glucose levels, a stress indicator in fish. Our results demonstrate that fish kept at higher water temperature without any probiotic supplementation have elevated blood glucose levels due to increased gluconeogenesis [32]. However, fish fed with probiotics showed lower blood glucose levels, which might be due to the capability of probiotics to reduce the effects of stressors. Probiotics have long been reported to be significantly and positively correlated with improved glucose tolerance, insulin secretion and normalized immune response [33].

The respiratory burst activity of phagocytes, measured by the reduction of NBT, was significantly decreased at higher temperatures, but increased when fish were fed a diet supplemented with probiotics. This increase in the respiratory burst activity in the probiotic fed fish could be due to the increased bacterial pathogen killing activity of phagocytes [34] and hence, better immune status of fish [6]. This was supported by elevated MPO activity in the probiotic supplemented groups. In a different study, Sakai [35] reported that the presence of probiotics, *Bacillus coagulans* B16 and *Rhodopseudomonas palustris* G06, were able to increase immune responses such as MPO and respiratory burst activities of tilapia [36], [37].

Studies show that incorporation of probiotics stimulates the hemopoesis and also induces the non specific immunity in fish [38]. Similarly, the RBC content of the probiotic supplemented fish groups was found to be higher than its non-supplemented counterparts. Dahiya et al. [39] reported an increase in the packed cell volumes of the blood as well as increased production of erythrocytes and leukocytes when fed with a diet incorporated with the probiotic, *Lactobacillus sporogens*. On the contrary, an increased WBC value, in response to high temperature was noticed in fish fed with a diet lacking probiotics [40], which however, decreased after probiotic supplementation, thus suggesting an improved immunity [16]. The reduction of WBC count might also be associated with anti-stress substances released/produced by probiotic organisms. In this regard, Matsumoto et al [41] reported that a strain of Bifidobacterium animalis can increase longevity in mice via its influence on gut polyamine production, an anti-stress substance which also reduces the WBC concentration in the blood [42].

Probiotics are responsible for enhanced immunoglobulin and antibody levels in the body by stimulating the lymphocyte proliferation (both B and T cells) [43], [44]. Dietary administration of a multispecies probiotic supplement increased the number of Ig cells in the sea bream [45] and gilthead seabream [46]. Concomitantly, in the present investigation, the serum IgM, level was found to increase after probiotic supplementation. However, temperature had an adverse effect on IgM production as well as serum globulin and total protein levels suggest possible deterioration of health. Enhancement of serum proteins and reduction of A:G ratio in the probiotic fed fish, in comparison to the non-probiotic fed fish, suggests the immune enhancing properties of probiotics [47]. We also observed reduced ALP and ACP activities in the probiotic fed groups. However, groups fed with non- probiotic diet showed higher ALP and ACP levels. An increase in the activity of these two enzymes with increasing temperature is indicative of cell damage in liver, intestine or kidney [47], [48].

Apoptosis can be considered as a contributor to the pathology of many diseases, including disorders of immune function [49]. In fathead minnow, *Pimephales promelas*, the gills showed maximum response to heat shock compared to other tissues [50]. We also observed similar results with higher apoptosis rate in gills than liver and kidney. The occurrence of a relatively higher amount of apoptosis-positive cells in the gills compared to other tissues might be due to the direct exposure to the external stressful environment [51]. Weber and Janz [52] reported that increased stress levels in the liver of juvenile catfish may protect the hepatic cells from apoptosis. Our results are in line with the findings of Weber and Janz [52] in case of liver, but not in kidney, which showed severe apoptosis at higher temperature of 37°C in the non-probiotic fed groups. This might be probably due to the failure of cellular defense mechanism under excessive stressful condition [53]. To our surprise, although significant differences in apoptotic cell counts were observed between probiotic-supplemented groups maintained at 28 and 37°C, histological differences were rare (data not shown). As our cell death analysis was based on the changes at transcriptional level, the positive cells only seem to progress towards apoptosis but were not necessarily disintegrated (the final step of apoptosis) (Figs. 2A–I), which could be easily reversed after stress reduction, in this case, with the probiotic-supplementation. This suggests that probiotic addition helps to withstand the effects of temperature stress quite effectively.

HSPs, temperature associated stress indicators, are expressed differently with varying temperature [18], [37]. Das et al [27] reported that an increase in temperature can linearly induce HSP production in *L. rohita* fingerlings. In the present study, an increase in temperature resulted in a significant increase in HSP70 levels, suggesting cross protection against lethal stressors (in this case: higher temperature) [27]. Significant reduction of HSP70 levels was observed in the probiotic fed groups in comparison to the non-probiotic fed counterparts. Stress reducing factors produced by probiotics [41] might have lowered the HSP levels in probiotic fed fish at higher temperatures and resulted in better growth and immunity.

The combination of a bacteria and yeast in the probiotic mixture resulted in higher growth rate and better survival in carp [54]. We have earlier reported that an increase in the body weight of *L. rohita* fingerlings after dietary multispecies probiotic supplementation is mostly due to the higher degree of adhesion of beneficial microbes and simultaneous reduction of total heterotrophic bacteria counts in the intestine [15]. In the present study, improved % weight gain noticed in the groups reared at higher temperature and fed with probiotic-supplemented diet could be due to the benefits of probiotic supplementation such as improvement in feed values, enhanced enzymatic digestion, higher metabolism, better nutrient utilization and production of growth promoting factors [15], [55]. In accordance with Das et al [23] we also recorded reduced growth at higher temperatures (>33°C). This reduction might be related to temperature associated anomalies which could be overcome (to a certain extent) by the probiotic supplementation.

Summarizing the above findings, dietary multispecies probiotic supplementation, with two bacterial species (*B. subtilis* and *L. lactis*) and one yeast (*S. cerevisiae*), resulted in enhanced growth and better hemato-immunological status of the *L. rohita* fingerlings reared at higher water temperature. Probiotic addition might have reduced the temperature associated stresses, which in turn lowered the apoptosis in probiotic fed fish. However, pond trials are necessary to implement this multispecies probiotic enhanced and nutritionally balanced diet in commercial tropical carp farms.
